# Complete Genome Sequence of a New Tobamovirus Naturally Infecting Tomatoes in Mexico

**DOI:** 10.1128/genomeA.00794-13

**Published:** 2013-10-03

**Authors:** Rugang Li, Shan Gao, Zhangjun Fei, Kai-Shu Ling

**Affiliations:** USDA–Agricultural Research Service, U.S. Vegetable Laboratory, Charleston, South Carolina, USAa; Boyce Thompson Institute for Plant Research, Cornell University, Ithaca, New York, USAb; USDA–Agricultural Research Service, Robert W. Holley Center for Agriculture and Health, Ithaca, New York, USAc

## Abstract

The complete genomic sequence of a new tobamovirus in tomatoes was determined through deep sequencing and assembly of small RNAs, then validated through Sanger sequencing. Based on the low sequence identity (≤85%) to known viruses and a close phylogenetic relationship to tobamoviruses, it was identified as a new species.

## GENOME ANNOUNCEMENT

*Tobamovirus* is the largest genus in the family *Virgaviridae* (1) and consists of 29 definitive and 6 unclassified species. Among tobamoviruses, tobacco mosaic virus (TMV) and tomato mosaic virus (ToMV) have been reported naturally infecting tomatoes. In the present study, we determined the complete genomic sequence of a new tobamovirus, provisionally named tomato mottle mosaic virus (ToMMV), which was isolated from a greenhouse tomato sample (MX5) collected in Mexico (Tamazula, Jalisco) in 2009. From the same sample, we identified a potyvirus in a previous study (2). ToMMV was biologically purified through three passages on the local lesion host *Nicotiana rustica* and maintained on the tomato “Money-maker” in a growth chamber at 25°C with a photoperiod of 14 h. In this study, the suspected new virus induced a rapid tissue necrosis on the upper leaves of inoculated tomato seedlings or mosaic and leaf distortion on mature plants. In order to determine the genomic sequence, we isolated total RNA from infected tomato leaves using Trizol reagents (Invitrogen). After separation of the small RNA (sRNA) from the total RNA by polyacrylamide gel electrophoresis, the sRNA library was constructed according to the published protocol (3) and sequenced in an Illumina HiSeq 2000. Finally, sRNA sequences were assembled based on the bioinformatics process as described previously (4). To confirm the identified ToMMV sequence, overlapping reverse transcription-PCR (RT-PCR) products covering the entire virus genome were generated using virus-specific primers and sequenced by use of Sanger technology. The 5′-terminal region was determined by rapid amplification of cDNA ends (RACE) technology using a FirstChoice RLM-RACE kit (Ambion). The 3′-terminal region was determined through the ligation of a miRNA linker (rAppCTGTAGGCACCATCAAT-amine; New England Biolabs) to the purified total RNA and followed by RT-PCR with a viral primer (5′-GCGATCAGGTCCGCTGTTAA-3′) and the reverse primer (5′-ATTGATGGTGCCTACAG-3′). The DNASTAR Lasergene 10 program (Madison, WI) was used to assemble and analyze the ToMMV sequence (6,398 nucleotides [nt]) from the Sanger sequencing. With this ToMMV genome sequence obtained by the Sanger technology as a reference, sRNAs generated on an Illumina HiSeq 2000 were reassembled to produce a full-length consensus genome sequence. Comparative analysis revealed that the consensus ToMMV and the Sanger sequencing-derived ToMMV sequence were nearly identical, with the exception of 3 single nucleotide polymorphisms at positions 898 (C or T versus C), 1522 (A or T versus T), and 3655 (G versus A).

A BLASTn search using the full genome sequence indicated that ToMMV was most closely related to ToMV (FN985165), with an identity of 85%. Phylogenetic analysis showed that ToMMV was clustered together with a group of tobamoviruses mainly infecting solanaceous plants and nested within the subclade of ToMV, TMV, and rehmannia mosaic virus. The criteria for *Tobamovirus* species demarcation stipulate that less than 90% genome sequence identity is considered a new species (5). Therefore, it is evident that ToMMV is a new species of the genus *Tobamovirus* in the family *Virgaviridae*.

### Nucleotide sequence accession number.

The full genomic sequence of ToMMV was deposited in GenBank under accession number KF477193.
